# Comorbidities and comorbidity burden in patients with neurocognitive disorders: findings from the MEMORA Cohort Study

**DOI:** 10.1007/s41999-025-01288-8

**Published:** 2025-08-27

**Authors:** Mohamed Nour Temedda, Antoine Garnier-Crussard, Claire Moutet, Christelle Mouchoux, Virginie Dauphinot

**Affiliations:** 1 Clinical and Research Memory Center of Lyon, Charpennes Hospital, Department of Geriatric Medicine, Lyon Institute For Aging, Hospices Civils de Lyon, Lyon 1 University, 27 rue Gabriel Péri, 69100 Villeurbanne, France; 2 INSERM U1028, CNRS UMR5292, Lyon Neuroscience Research Center, Brain Dynamics and Cognition Team, University Lyon 1, 69000 Lyon, France; 3 Clinical Research Center Ageing-Brain-Frailty, University Claude Bernard Lyon 1, Hospices Civils de Lyon, 69100 Villeurbanne, France; 4 Normandie Univ, UNICAEN, INSERM, U1237, PhIND “Physiopathology and Imaging of Neurological Disorders”, NeuroPresage Team, Institute Blood and Brain @ Caen-Normandie, Cyceron, 14000 Caen, France; 5 Pharmaceutical Unit, Charpennes Hospital, University Hospital of Lyon, 69100 Villeurbanne, France

**Keywords:** Comorbidity indices, Comorbidities, Neurocognitive disorders, Dementia, MCI

## Abstract

**Aim:**

This study aimed to compare comorbidities’ prevalence and the comorbidity burden as measured by comorbidity indices according to the stages and the etiological diagnoses of neurocognitive disorders (NCD). This study aimed also to examine how comorbidities and comorbidity indices were associated with both stages and etiologies of NCD.

**Methods:**

This is a cross-sectional study including participants from the MEMORA real-life cohort, aged ≥ 60 years. Patients had to be in either MCI or dementia stage, with the following etiological diagnoses of NCD: AD, VD (vascular dementia), mixed dementia (MD), dementia with Lewy bodies, frontotemporal dementia and Parkinson’s disease. Three comorbidity indices were used to measure comorbidity burden: the Charlson comorbidity index, the multimorbidity-weighted index (MWI) and the health-related quality of life comorbidity index (HRQOL-CI). Prevalence of comorbidities involved in the calculation of at least one comorbidity index were reported. The associations between comorbidities/comorbidity indices and groups were assessed using binary and multinomial logistic regressions.

**Results:**

Overall, 3470 patients (66.9% dementia; 50.9% AD) were included. The most frequent comorbidities were hypertension (50.2%), depression (28.0%) and hypercholesterolemia (22.6%). The MWI and the HRQOL-CI were associated with increased odds of dementia (vs. MCI). Upon stratifying population according to the etiological diagnoses of NCD, the highest mean of the three comorbidity indices were found in patients with VD and MD.

**Conclusion:**

Comorbidities and comorbidity burden vary according to the stage and etiological diagnoses of NCD. Future studies should consider the stage and the etiological diagnoses, when studying the effect of the comorbidity burden on the progression of NCD.

**Supplementary Information:**

The online version contains supplementary material available at 10.1007/s41999-025-01288-8.

## Key summary points

**Aim:**

This study aimed to compare comorbidities’ prevalence and the comorbidity burden as measured by comorbidity indices according to the stages and the etiological diagnoses of neurocognitive disorders (NCD). This study aimed also to examine how comorbidities and comorbidity indices were associated with both stages and etiologies of NCD.

**Findings:**

Higher comorbidity burden was found in patients with dementia compared to those with mild cognitive impairment. Higher comorbidity burden was found in vascular dementia and mixed dementia compared to other etiological diagnoses of NCD.

**Message:**

Comorbidities and comorbidity burden vary according to the stage and etiological diagnoses of NCD.

**Supplementary Information:**

The online version contains supplementary material available at 10.1007/s41999-025-01288-8.

## Introduction

The global prevalence of dementia was approximately 55 million as estimated in 2019, and it is expected to triple by 2050 [1]. Alzheimer’s disease (AD) is the most frequent etiological diagnosis of dementia followed by vascular dementia (VD) [2]. The lack of curative treatment for these neurocognitive disorders (NCD) has increased interest to identify and manage modifiable risk factors such as comorbidity burden [3].

Previous studies have used comorbidity indices to assess comorbidity burden in individuals with NCD and to examine its impact on outcomes such as cognitive decline and functional decline [4, 5]. Nevertheless, findings (association between comorbidity indices and cognitive decline/functional decline) were inconsistent between studies which may be related to the heterogeneity of the population with NCD [4, 5]. Higher comorbidity burden as measured by the Charlson comorbidity index (CCI) [6, 7] and the cumulative illness rating scale-geriatric (CIRS-G) [8] was found in patients with dementia compared to those with mild cognitive impairment (MCI) [6–8]. Regarding etiological diagnoses of NCD, a higher CCI was found in patients with VD than in patients with AD [9]. Other studies had compared some specific comorbidities and findings were as following: a higher prevalence of hypertension, diabetes and depression in patients with dementia compared to those with MCI [6, 7, 10]. Regarding etiologic diagnoses, patients with VD have a higher prevalence of cardiovascular diseases compared to those with AD [9]. The comparison between dementia with Lewy bodies (DLB) and AD indicated that patients with DLB presented a higher prevalence of various comorbidities, including diseases of the nervous system, diseases of the connective tissue as well as disorders affecting the circulatory, respiratory, and genitourinary systems [11].

Comorbidity burden as measured by comorbidity indices appears to vary according to NCD stage and etiology. This variation may be explained by comorbidities’ distribution according NCD stage and etiology, since that comorbidity indices consider number and severity of comorbidities [12]. In this context, the majority of studies have compared the comorbidities’ prevalence, or/and the comorbidity indices between patients with MCI and dementia. Nevertheless, studies that had considered etiological diagnoses of NCD, focused on a few etiological diagnoses such as AD and VD. Thus, there is a lack of studies that consider simultaneously other etiological diagnoses such as mixed dementia (MD), DLB, frontotemporal dementia (FTD) and Parkinson’s disease (PD) to compare the comorbidities’ prevalence, and comorbidity indices.

While several comorbidity indices have been developed, they vary in both their complexity and the type of data required. In this study, alongside a detailed identification of individual comorbidities, we focused on three scores i.e. the CCI, the Multimorbidity-Weighted Index (MWI), and the Health-Related Quality of Life Comorbidity Index (HRQOL-CI) that could be calculated using information routinely collected in clinical practice based on available data and that were considered relevant in older populations and individuals with NCD to the patients with NCD, as supported by the literature [13–15]. These indices were shown to significantly predict a range of clinically relevant outcomes, including cognitive and functional decline, institutionalization, and mortality. Given their established prognostic value, understanding which comorbidities are most common, and how they differ across stages and etiologies of NCD, can help to identify vulnerable patient subgroups, tailor clinical management strategies, and improve prognostic evaluation in individuals with NCD.

This study aimed to compare comorbidities’ prevalence and the comorbidity burden as measured by the CCI, the MWI and the HRQOL-CI according to the stage of NCD (i.e. MCI/dementia), and the following etiological diagnoses of NCD (AD, MD, VD, DLB, FTD, and PD). This study aimed also to examine how comorbidities and comorbidity indices were associated with both stages and etiologies of NCD.

## Methods

### Study design and setting

This is a cross-sectional study including participants from the MEMORA real-life cohort. The MEMORA cohort includes outpatients attending a memory consultation and was conducted in the clinical and research memory center of Lyon, France [16]. The MEMORA cohort protocol (clinicaltrial.gov number NCT02302482) has been approved by the regional ethics committee (Comité de protection des personnes Sud Est III) on July 29, 2014. Data processing has been approved by the national data protection commission.

### Population

The patients included in the present study were required to be aged 60 years or older, with the following etiological diagnoses of NCD: AD [17], VD [18], mixed dementia (AD with cerebrovascular disease) [17, 18], LBD [19], FTD [20] and PD [21]. Patients had to be either in the stage of MCI or dementia. The etiological diagnosis and the stage of NCD were identified through the medical evaluation of the geriatrician, neurologist or psychiatrist in charge of the patients. MCI and dementia stages were identified using the Diagnosis and Statistical Manual of mental disorders (DSM-V) [17, 22, 23]. Patients with incomplete diagnostic information or with a missing or undetermined etiological diagnosis of NCD were excluded. This study includes patients recruited between 2014 and 2022.

### Comorbidities, comorbidity burden and patients’ characteristics

Comorbidities were collected during the first memory consultation by the specialist physician from the patients and their caregivers (if present) through an open-ended question asking to list the past and present comorbidities of the patients, completed with the information in the letter of patients’ general practitioner. Patients were considered to have or not have each of these comorbidities, regardless of the date of diagnosis as it was not available. The comorbidities of interest were selected according to those used in the calculation of the three comorbidity indices also examined in this study to measure comorbidity burden [14]: the CCI, the MWI or the HRQOL-CI.

The comorbidity indices were computed by identifying specific comorbidities and summing weights attributed to each comorbidity (supplementary Table 1) [24–26]: For the CCI the weights of comorbidities were based on their associations with 1-year mortality prediction in hospitalized patients [24], for MWI the weights of comorbidities were based on their association with short form-36 physical functioning [15, 25], and for the HRQOL-CI the weights of comorbidities were based on their association with the short form-12 physical component summary [26]. Dementia or etiological diagnosis of NCD were not considered to calculate the three comorbidity indices as they correspond to the primary condition of included patients.

The other characteristics considered in this study were the age, the sex and the educational level (till/primary/secondary/tertiary) of the patients collected at the first consultation. All the data were collected in a eCRF by trained physicians, nurses and research assistants.

### Statistical analysis

Prevalence of comorbidities was described by percentage with 95% confidence interval (95%CI) calculated using the Wilson equation [27]. Comorbidity indices considered as quantitative variables were described using mean ± standards deviation (SD) and median, 1 st and 3rd quartiles. Prevalences were compared between groups (stages and etiological diagnoses of NCD) using Pearson’s chi-squared test or Fisher's exact test. When a significant difference in comorbidities was observed between groups, prevalence rates were adjusted for age, sex, and education level (these factors are known to influence both the prevalence of comorbidities and cognitive outcomes [11, 28, 29]; see supplementary Table 2). In addition, adjusted prevalences were derived from the logistic models using marginal standardization. The associations between comorbidities and groups were assessed using binary (stages of NCD with MCI as reference category) and multinomial (etiological diagnoses with AD as reference category) logistic regressions. Adjusted odds ratios (OR) with their 95%CI were reported to quantify the strength of these associations while controlling for confounders (age, sex and education level).

Comorbidity indices were compared between groups using Student’s t-test or ANOVA. Means of comorbidity burden indices were also adjusted for age, sex and education level using ANOVA model. Additional analyses were also performed to compare between AD and others NCD etiologies (MD, VD, DLB, FTD and PD; with AD as reference category; supplementary Table 3). P-values (p) and adjusted p (correction for multiple comparisons using method reported by Benjamini-Hochberg) lower than 0.05 were considered as statistically significant. Statistical analyses were performed with R statistical software (version R.4.4.0; Lyon1, France).

## Results

As shown in Table 1, 3470 patients were included, with a mean age of 82.9 ± 5.5 years, and 60.4% (n = 2096) of females. The majority of patients were at dementia stage (66.9%) and with AD diagnosis (n = 1765, 50.9%). The proportion of the others etiological diagnoses was as follow: MD (n = 894, 25.8%), VD (n = 494, 14.2%), DLB (n = 153, 4.4%), FTD (n = 83, 2.4%) and PD (n = 81, 2.3%).

**Table 1:** Sociodemographic characteristics of patients

	Total sample (N = 3470)	Stage of NCD		p-value	Etiological diagnoses of NCD						p-value
		Dementia (n = 2321, 66.9%)	MCI (n = 1149, 33.1%)		AD (n = 1765, 50.9%)	MD (n = 894, 25.8%)	VD (n = 494, 14.2%)	DLB (n = 153, 4.4%)	FTD (n = 83, 2.4%)	PD (n = 81, 2.3%)	
Age, year, mean ± SD	82.9 ± 6.9	83.3 ± 6.9	82.2 ± 6.7	< 0.001	83.0 ± 7.0	84.7 ± 5.7	81.8 ± 7.1	80.9 ± 6.6	74.6 ± 6.3	80.5 ± 5.7	< 0.001
Age category, n (%)											
≥ 80 years	2393 (68.9)	1663 (71.6)	730 (63.5)	< 0.001	1219 (69.1)	715 (80)	307 (62.2)	89 (58.2)	19 (22.9)	44 (54.3)	< 0.001
Sex/gender, n (%)											
Female	2096 (60.4)	1426 (61.4)	670 (58.3)	0.08	1171 (66.3)	544 (60.8)	243 (49.2)	76 (49.7)	37 (44.6)	25 (30.9)	< 0.001
Education level, n (%)				< 0.001							< 0.001
Till	674 (19.4)	539 (23.2)	135 (11.7)		329 (19.2)	189 (21.1)	104 (21.1)	36 (23.5)	9 (10.8)	7 (8.6)	
Primary	1114 (32.1)	778 (33.5)	336 (29.2)		595 (34.7)	302 (33.8)	150 (30.4)	33 (21.6)	20 (24.1)	14 (17.3)	
Secondary	1117 (32.2)	684 (29.5)	433 (36.7)		564 (32.9)	289 (32.3)	144 (29.1)	60 (39.2)	29 (34.9)	31 (38.3)	
Tertiary	565 (16.3)	320 (13.8)	245 (21.3)		227 (13.2)	114 (12.8)	96 (19.4)	24 (15.7)	25 (30.1)	29 (35.8)	

*NCD* Neurocognitive disorders, *AD* Alzheimer’s disease, *MD* Mixed dementia, *VD* Vascular dementia, *DLB* Dementia with Lewy bodies, *FTD* Frontotemporal dementia, *PD* Parkinson’s disease, *MCI* Mild cognitive impairment

### Comorbidities’ distribution in the total sample

In the total sample, the most prevalent comorbidities were hypertension (50.2%), depression (28%), hypercholesterolemia (22.6%), vision disorders (22.1%), chronic obstructive pulmonary diseases/Asthma (21.7%), diabetes (20.6%), peripheral vascular diseases (19.1%), all cancer cases (19.4%), and congestive heart failure (17.8%) (Table 2).

**Table 2:** Prevalence of comorbidities in dementia and MCI

Comorbidities	Stage of NCD						
	Total sample (N = 3470)		Dementia (n = 2321, 66.9%)		MCI (n = 1149, 33.1%)		
	n	% (95% CI)	n	% (95% CI)	n	% (95% CI)	p-value
Myocardial							
CHF	617	17.8 (16.5–19.1)	466	20.1 (18.5–21.8)	151	13.1 (11.3–15.2)	< 0.001
Arrhythmia	589	17.0 (15.8–18.3)	408	17.6 (16.1–19.2)	181	15.8 (13.8–18.0)	0.19
Valvular diseases	130	3.7 (3.2–4.4)	91	3.9 (3.2–4.8)	39	3.4 (2.5–4.6)	0.50
Angina	39	1.1 (0.8–1.5)	29	1.2 (0.9–1.8)	10	0.9 (0.5–1.6)	0.41
MI	108	3.1 (2.6–3.7)	76	3.3 (2.6–4.1)	32	2.8 (2.0–3.9)	0.50
Ischemic heart diseases	179	5.2 (4.5–5.9)	116	5.0 (4.2–6.0)	63	5.5 (4.3–7.0)	0.60
Vascular							
Hypertension	1742	50.2 (48.5–51.9)	1194	51.4 (49.4–53.5)	548	47.7 (44.8–50.6)	0.04
Hypercholesterolemia	784	22.6 (21.2–24.0)	500	21.5 (19.9–23.3)	284	24.7 (22.3–27.3)	0.04
PVD	664	19.1 (17.9–20.5)	453	19.5 (18.0–21.2)	211	18.4 (16.2–20.7)	0.44
Anemia	234	6.7 (6.0–7.6)	186	8.0 (7.0–9.2)	48	4.2 (3.2–5.5)	< 0.001
Endocrine							
Diabetes	716	20.6 (19.3–22.0)	507	21.8 (20.2–23.6)	209	18.2 (16.1–20.5)	0.011
Uncomplicated diabetes	518	14.9 (13.8–16.2)	363	15.6 (14.2–17.2)	155	13.5 (11.6–15.6)	0.10
Complicated diabetes	198	5.7 (5.0–6.5)	144	6.2 (5.3–7.3)	54	4.7 (3.6–6.1)	0.08
Thyroid disorders	422	12.2 (11.1–13.3)	279	12.0 (10.8–13.4)	143	12.4 (10.7–14.5)	0.76
Neurological							
CVD	478	13.8 (12.7–15.0)	286	12.3 (11.0–13.7)	192	16.7 (14.7–19.0)	< 0.001
Hemiplegia	35	1.0 (0.7–1.4)	23	1.0 (0.7–1.5)	12	01.0 (0.6–1.8)	0.99
Spinal cord disorders	236	6.8 (6.0–7.7)	152	6.5 (5.6–7.6)	84	7.3 (5.9–9.0)	0.44
Psychiatric							
Depression	973	28.0 (26.6–29.6)	667	28.7 (26.9–30.6)	306	26.6 (24.2–29.3)	0.21
Renal							
Chronic renal diseases	484	13.9 (12.8–15.1)	339	14.6 (13.2–16.1)	145	12.6 (10.8–14.7)	0.12
Pulmonary							
COPD/Asthma	754	21.7 (20.4–23.1)	507	21.8 (20.2–23.6)	247	21.5 (19.2–24.0)	0.85
Cancer							
Total Cancer	674	19.4 (18.1–20.8)	444	19.1 (17.6–20.8)	230	20.0 (17.8–22.4)	0.56
Non-Metastatic	640	18.4 (17.2–19.8)	419	18.1 (16.5–19.7)	221	19.2 (17.1–21.6)	0.42
Metastatic solid tumor	34	1.0 (0.7–01.4)	25	1.1 (0.7–1.6)	9	0.8 (0.4–1.5)	0.52
Liver							
Mild	71	2.0 (1.6–2.6)	41	1.8 (1.3–2.4)	30	2.6 (1.8–3.7)	0.13
Moderate to severe	43	1.2 (0.9–1.7)	25	1.1 (0.7–1.6)	18	1.6 (1.0–2.5)	0.29
Hepatitis	64	1.8 (1.4–2.3)	37	1.6 (1.2–2.2)	27	2.3 (1.6–3.4)	0.15
Connective							
Arthritis	168	4.8 (4.2–5.6)	125	5.4 (4.5–6.4)	43	3.7 (2.8–5.0)	0.041
Osteoporosis	316	9.1 (8.2–10.1)	222	9.6 (8.4–10.8)	94	8.2 (6.7–9.9)	0.20
Osteoarthritis	548	15.8 (14.6–17.0)	360	15.5 (14.1–17.0)	188	16.4 (14.3–18.6)	0.55
Hip replacement	242	7.0 (6.2–7.9)	163	7.0 (6.1–8.1)	79	6.9 (5.6–8.5	0.93
Knee replacement	106	3.1 (2.5–3.7)	76	3.3 (2.6–4.1)	30	2.6 (1.8–3.7)	0.33
Ophthalmologic							
Vision disorders	766	22.1 (20.7–23.5)	523	22.5 (20.9–24.3)	243	21.1 (18.9–23.6)	0.38
Infections/Immune							
AIDS	4	0.1 (0.0–0.3)	3	0.1 (0.0–0.4)	1	0.1 (0.0–0.5)	1*
Digestives							
Gastric ulcer	240	6.9 (6.1–07.8)	167	7.2 (6.2–8.3)	73	6.4 (5.1–7.9)	0.39
Esophageal disorders	38	1.1 (0.8–1.5)	22	0.9 (0.6–1.4)	16	1.4 (0.9–2.3)	0.31

*NCD* Neurocognitive disorders, *CHF* Congestive heart failure, *MI* Myocardial infraction, *PVD* Peripheral vascular diseases, *CVD* Cerebrovascular diseases, *COPD* Chronic obstructive pulmonary diseases, *CTD* Connective tissue diseases, *AIDS* Acquired immune deficiency syndrome, *MCI* Mild cognitive impairment, *CI* Confidence interval, *p* p-value

^*^Fisher's exact test

### Comorbidities according to the stage of NCD

Five comorbidities were significantly more prevalent in patients with dementia compared to patients with MCI: congestive heart failure (20.1% vs. 13.1%), hypertension (51.4% vs. 47.7%), anemia (8% vs. 4.2%), diabetes (21.8% vs. 18.2%) and arthritis (5.4% vs. 3.7%; Table 2). After adjustment (adjustment for age, sex and educational level because these variables are known to influence both the prevalence of comorbidities and cognitive outcomes), congestive heart failure, anemia, diabetes and arthritis were associated with increased odds of dementia compared to MCI (Table 4). In contrast, the difference in hypertension did not remain significant between MCI and dementia after adjustment. Two comorbidities were significantly higher in patients with MCI compared to patients with dementia: hypercholesterolemia (24.7% vs. 21.5%) and cerebrovascular diseases (16.7% vs. 12.3%; Table 3). These findings remained similar after adjustment (Table 4).

**Table 3:** Prevalence of comorbidities according to etiological diagnoses of NCD

Comorbidities	Etiological diagnoses of NCD												
	AD (n = 1765, 50.9%)		MD (n = 894, 25.8%))		VD (n = 494, 14.2%)		DLB (n = 153, 4.4%)		FTD (n = 83, 2.4%)		PD (n = 81, 2.3%)		p-value
	n	% (95%CI)	n	% (95%CI)	n	% (95%CI)	n	% (95%CI)	n	% (95%CI)	n	% (95%CI)	
Myocardial													
CHF	289	16.4 (14.7–18.2)	179	20.0 (17.5–22.8)	104	21.1 (17.7–24.9)	28	18.3 (13.0–25.2)	7	8.4 (4.1–16.4)	10	12.3 (6.8–21.3)	0.008
Arrhythmia	261	14.8 (13.2–16.5)	183	20.5 (18.0–23.2)	13	20.9 (17.5–24.7)	27	17.6 (12.4–24.5)	8	9.6 (5.0–17.9)	7	8.6 (4.2–16.8)	< 0.001
Valvular diseases	63	3.6 (2.8–4.5)	50	5.7 (4.4–7.4)	14	3.0 (1.8–4.9)	1	0.7 (0.1–3.6)	0	-	2	2.5 (0.7–8.6)	0.004*
Angina	17	1.0 (0.6–1.5)	12	1.3 (0.8–2.3)	6	1.2 (0.6–2.6)	2	1.3 (0.4–4.6)	1	1.2 (0.2–6.5)	1	1.2 (0.2–6.7)	0.85
MI	48	2.7 (2.1–3.6)	37	4.1 (3.0–5.7)	11	2.2 (1.2–3.9)	6	3.9 (1.8–8.3)	2	2.4 (0.7–8.4)	4	4.9 (1.9–12.0)	0.20
Ischemic heart diseases	82	4.6 (3.8–5.7)	55	6.2 (4.8–7.9)	26	5.3 (3.6–7.6)	9	5.9 (3.1–10.8)	3	3.6 (1.2–10.1)	4	4.9 (1.9–12.0)	0.65*
Vascular													
Hypertension	816	46.2 (43.9–48.6)	512	57.3 (54.0–60.5)	282	57.1 (52.7–61.4)	69	45.1 (37.4–53.0)	30	36.1 (26.6–46.9)	33	40.7 (30.7–51.6)	< 0.001
Hypercholesterolemia	378	21.4 (19.6–23.4)	201	22.5 (19.9–25.3)	139	28.1 (24.4–32.3)	35	22.9 (16.9–30.1)	18	21.7 (14.2–31.7)	13	16.0 (9.6–25.5)	0.033
PVD	293	16.6 (14.9–18.4)	202	22.6 (20.0–25.5)	107	21.7 (18.3–25.5)	34	22.2 (16.4–29.4)	15	18.1 (11.3–27.7)	13	16.0 (9.6–25.5)	0.003
Anemia	105	05.9 (04.9–07.2)	67	7.5 (5.9–9.4)	36	7.3 (5.3–9.9)	10	6.5 (3.6–11.6)	7	8.4 (4.1–16.4)	9	11.1 (6.0–19.8)	0.34
Endocrine													
Diabetes	294	16.7 (15.0–18.5)	204	22.8 (20.2–25.7)	143	28.9 (25.1–33.1)	38	24.8 (18.7–32.2)	19	22.9 (15.2–33.0)	18	22.2 (14.5–32.4)	< 0.001
Uncomplicated diabetes	223	12.6 (11.2–14.3)	140	15.7 (13.4–18.2)	101	20.4 (17.1–24.2)	27	17.6 (12.4–24.5)	16	19.3 (12.2–29.0)	11	13.6 (7.8–22.7)	< 0.001
Complicated diabetes	71	4.0 (3.2–5.0)	64	7.2 (5.6–9.0)	42	8.5 (6.4–11.3)	11	7.2 (4.1–12.4)	3	3.6 (1.2–10.1)	7	8.6 (4.2–16.8)	0.002*
Thyroid disorders	233	13.2 (11.7–14.9)	97	10.9 (9.0–13.1)	58	11.7 (9.2–14.9)	17	11.1 (7.1–17.1)	8	9.6 (5.0–17.9)	9	11.1 (6.0–19.8)	0.54
Neurological													
CVD	114	6.5 (5.4–7.7)	221	24.7 (22.0–27.7)	117	23.7 (20.1–27.6)	17	11.1 (7.1–17.1)	3	3.6 (1.2–10.1)	6	7.4 (3.4–15.2)	< 0.001*
Hemiplegia	14	0.8 (0.5–1.3)	11	1.2 (0.7–2.2)	6	1.2 (0.6–2.6)	1	0.7 (0.1–3.6)	1	1.2 (0.2–6.5)	3	3.7 (1.3–10.3)	0.21*
Spinal cord disorders	90	5.1 (4.2–6.2)	77	8.6 (6.9–10.6)	35	7.1 (5.1–9.7)	16	10.5 (6.5–16.3)	4	4.8 (1.9–11.7)	14	17.3 (10.6–26.9)	< 0.001*
Psychiatric													
Depression	543	30.8 (28.7–33.0)	199	22.3 (19.7–25.1)	132	26.7 (23.0–30.8)	46	30.1 (23.4–37.7)	33	39.8 (29.9–50.5)	20	24.7 (16.6–35.1)	< 0.001
Renal													
Chronic renal diseases	234	13.3 (11.8–14.9)	132	14.8 (12.6–17.2)	84	17.0 (13.9–20.6)	20	13.1 (8.6–19.3)	8	9.6 (5.0–17.9)	6	7.4 (3.4–15.2)	0.10
Pulmonary													
COPD/Asthma	367	20.8 (19.0–22.7)	202	22.6 (20.0–25.5)	122	24.7 (21.1–28.7)	32	20.9 (15.2–28.0)	19	22.9 (15.2–33.0)	12	14.8 (8.7–24.1)	0.28
Cancer													
Total Cancer	328	18.6 (16.8–20.5)	175	19.6 (17.1–22.3)	107	21.7 (18.3–25.5)	29	19.0 (13.5–25.9)	20	24.1 (16.2–34.3)	15	18.5 (11.6–28.3)	0.61
Non-Metastatic	312	17.7 (16.0–19.5)	161	18.0 (15.6–20.7)	104	21.1 (17.7–24.9)	29	19.0 (13.5–25.9)	19	22.9 (15.2–33.0)	15	18.5 (11.6–28.3)	0.53
Metastatic solid tumor	16	0.9 (0.6–1.5)	14	1.6 (0.9–2.6)	3	0.6 (0.2–1.8)	0	-	1	1.2 (0.2–6.5)	0	0.0 (0.0–4.5)	0.35*
Liver													
Mild	33	1.9 (1.3–2.6)	14	1.6 (0.9–2.6)	14	2.8 (1.7–4.7)	4	2.6 (1.0–6.5)	4	4.8 (1.9–11.7)	2	2.5 (0.7–8.6)	0.21*
Moderate to severe	17	1.0 (0.6–1.5)	10	1.1 (0.6–2.0)	12	2.4 (1.4–4.2)	3	2.0 (0.7–5.6)	1	1.2 (0.2–6.5)	0	-	0.13*
Hepatitis	28	1.6 (1.1–2.3)	13	1.5 (0.9–2.5)	13	20.9 (17.5–24.7)	4	2.6 (1.0–6.5)	4	4.8 (1.9–11.7)	2	2.5 (0.7–8.6)	0.12*
Connective													
Arthritis	79	4.5 (3.6–5.5)	44	4.9 (3.7–6.5)	33	6.7 (4.8–9.2)	7	4.6 (2.2–9.1)	2	2.4 (0.7–8.4)	3	3.7 (1.3–10.3)	0.45*
Osteoporosis	172	9.7 (8.4–11.2)	84	9.4 (7.7–11.5)	38	7.7 (5.7–10.4)	15	9.8 (6.0–15.5)	2	2.4 (0.7–8.4)	5	6.2 (2.7–13.6)	0.18
Osteoarthritis	272	15.4 (13.8–17.2)	155	17.3 (15.0–20.0)	85	17.2 (14.1–20.8)	19	12.4 (8.1–18.6)	8	9.6 (5.0–17.9)	9	11.1 (6.0–19.8)	0.18
Hip replacement	119	6.8 (5.7–8.1)	62	6.9 (5.4–8.8)	36	7.3 (5.3–9.9)	10	6.5 (3.6–11.6)	5	6.0 (2.6–13.3)	10	12.3 (6.8–21.3)	0.33*
Knee replacement	53	3.0 (2.3–3.9)	28	3.1 (2.2–4.5)	15	3.0 (1.8–4.9)	7	4.6 (2.2–9.1)	0	0.0 (0.0–4.4)	3	3.7 (1.3–10.3)	0.52*
Ophthalmologic													
Vision disorders	370	21.0 (19.1–22.9)	229	25.6 (22.9–28.6)	109	22.1 (18.6–25.9)	29	19.0 (13.5–25.9)	7	8.4 (4.1–16.4)	22	27.2 (18.7–37.7)	0.002
Infections/Immune													
AIDS	2	0.1 (0.0–0.4)	1	0.1 (0.0–0.6)	1	0.2 (0.0–1.1)	0	-	0	-	0	-	0.86*
Digestives													
Gastric ulcer	103	5.8 (4.8–7.0)	73	8.2 (6.5–10.1)	38	7.7 (5.7–10.4)	14	9.2 (5.5–14.8)	5	6.0 (2.6–13.3)	7	8.6 (4.2–16.8)	0.19
Esophageal disorders	19	1.1 (0.7–1.7)	10	1.1 (0.6–2.0)	6	1.2 (0.6–2.6)	1	0.7 (0.1–3.6)	1	1.2 (0.2–6.5)	1	1.2 (0.2–6.7)	0.98*

*CHF* Congestive heart failure, *MI* Myocardial infraction, *PVD* Peripheral vascular diseases, *CVD* Cerebrovascular diseases, *COPD* Chronic obstructive pulmonary diseases, *AIDS* Acquired immune deficiency syndrome, *AD* Alzheimer’s disease, *MD* Mixed dementia, *VD* Vascular dementia, *DLB* Dementia with Lewy bodies, *FTD* Frontotemporal dementia, *PD* Parkinson’s disease, *CI* Confidence interval, *p* p-value

* Fisher’s exact

**Table 4:** Adjusted OR of the association between comorbidities and either stage of etiological diagnoses of NCD

	Dementia vs. MCI (ref)		MD vs. AD (ref)		VD vs. AD (ref)		DLB vs. AD (ref)		FTD vs. AD (ref)		PD vs. AD (ref)	
	Adjusted OR (95%IC)	p-value	Adjusted OR (95%IC)	Adjusted p-value	Adjusted OR (95%IC)	Adjusted p-value	Adjusted OR (95%IC)	Adjusted p-value	Adjusted OR (95%IC)	Adjusted p-value	Adjusted OR (95%IC)	Adjusted p-value
Comorbidities												
CHF	1.53 (1.25–1.89)	< 0.001	1.11 (0.90–1.37)	0.41	1.31 (1.02–1.70)	0.18	1.15 (0.74–1.78)	0.53	0.61 (0.27–1.38)	0.40	0.70 (0.35–1.40)	0.41
Arrhythmia			1.37 (1.11–1.69)	0.008	1.52 (1.17–1.96)	0.008	1.27 (0.81–1.97)	0.37	0.79 (0.37–1.89)	0.54	0.55 (0.25–1.21)	0.26
Valvular diseases			1.59 (1.08–2.33)	0.046	0.83 (0.46–1.50)	0.63	0.20 (0.03–1.44)	0.18			0.70 (0.17–2.97)	0.63
Hypertension	1.05 (0.91–1.22)	0.48	1.50 (1.27–1.77)	< 0.001	1.64 (1.34–2.01)	< 0.001	1.03 (0.73–1.44)	0.89	0.92 (0.57–1.48)	0.10	0.97 (0.61–1.54)	0.99
Hypercholesterolemia	0.82 (0.70–0.98)	0.03	1.10 (0.90–1.34)	0.64	1.47 (1.70–1.85)	0.005	1.39 (0.77–1.69)	0.64	1.14 (0.65–1.97)	0.65	0.76 (0.41–1.40)	0.62
PVD			1.40 (1.46–1.72)	0.005	1.41 (1.09–1.81)	0.19	1.51 (1.01–2.27)	0.07	1.49 (0.82–2.70)	0.23	1.01 (0.55–1.87)	0.97
Anemia	1.88 (1.36–2.63)	< 0.001										
Diabetes	1.14 (0.95–1.37)	0.17	1.51 (1.23–1.86)	< 0.001	1.86 (1.46–2.36)	< 0.001	1.48 (0.99–2.20)	0.10	1.42 (0.82–2.45)	0.21	1.43 (0.82–2.48)	0.21
Uncomplicated diabetes			1.30 (1.02–1.64)	0.08	1.58 (1.21–2.07)	0.004	1.30 (0.83–2.04)	0.31	1.51 (0.84–2.72)	0.27	1.02 (0.53–1.99)	0.94
Complicated diabetes			1.86 (1.31–2.65)	0.001	2.11 (1.41–3.14)	0.001	1.75 (0.90–3.40)	0.12	0.94 (0.28–3.11)	0.92	2.51 (1.09–5.40)	0.049
CVD	0.70 (0.56–0.85)	< 0.001	4.72 (3.70–6.04)	< 0.001	4.27 (3.22–5.68)	< 0.001	1.70 (0.99–2.92)	0.09	0.45 (0.14–1.48)	0.24	1.01 (0.43–2.38)	0.98
Spinal cord disorders			1.69 (1.23–2.33)	0.003	1.46 (0.97–2.20)	0.08	2.34 (1.27–3.94)	0.008	1.23 (0.43–3.51)	0.70	4.41 (2.34–8.30)	< 0.001
Depression			0.68 (0.56–0.82)	< 0.001	0.87 (0.69–1.09)	0.39	1.02 (0.71–1.47)	0.89	1.39 (0.86–2.22)	0.39	0.78 (0.46–1.33)	0.45
Arthritis	1.50 (1.05–2.16)	0.028										
Vision disorders			1.22 (1.01–1.48)	0.13	1.15 (0.90–1.47)	0.34	0.99 (0.65–1.52)	0.98	0.52 (0.23–0.15)	0.17	1.66 (0.99–2.79)	0.13
Comorbidity indices												
CCI	1.02 (0.98–1.07)	0.23	1.17 (1.12–1.23)	< 0.001	1.23 (1.59–1.30)	< 0.001	1.11 (1.01–1.22)	0.06	1.11 (0.70–1.27)	0.16	0.95 (0.82–1.11)	0.54
MWI	1.02 (1.01–1.03)	0.04	1.05 (1.04–1.07)	< 0.001	1.07 (1.06–1.09)	< 0.001	1.03 (1.01–1.07)	0.07	1.01 (0.96–1.06)	0.78	0.99 (0.94–1.04)	0.78
HRQOL-CI	1.03 (1.01–1.06)	0.04	1.07 (1.04–1.10)	< 0.001	1.11 (1.07–1.15)	< 0.001	1.07 (1.01–1.39)	0.037	1.03 (0.94–1.13)	0.50	1.03 (0.95–1.12)	0.50

*CHF* Congestive heart failure, *PVD* Peripheral vascular diseases, *CVD* Cerebrovascular diseases, *AD* Alzheimer’s disease, *MD* Mixed dementia, *VD* Vascular dementia, *DLB* Dementia with Lewy bodies, *FTD* Frontotemporal dementia, *PD* Parkinson’s disease, *MCI* Mild cognitive impairment, *CCI* Charlson comorbidity index, *MWI* Multimorbidity-weighted index, *HRQOL*-*CI* Health related quality of life comorbidity index, *ref* reference category

### Comorbidities according to the etiological diagnoses of NCD

Hypertension was one of the most frequent comorbidities whatever the diagnoses of NCD. The others frequent comorbidities were depression in AD and DLB, vision disorders in MD and PD, cerebrovascular diseases in MD and diabetes in VD (Table 3). Thirteen comorbidities were significantly different according to the etiological diagnoses of NCD: congestive heart failure, arrythmia, valvular diseases, hypertension, hypercholesterolemia, peripheral vascular diseases, diabetes, uncomplicated diabetes, complicated diabetes, cerebrovascular diseases, spinal cord disorders, depression, and vision disorders.

Based on the adjusted multinomial logistic regressions (AD was the reference), congestive heart failure and hypercholesterolemia were associated with increased odds of VD, valvular diseases and vision disorders were associated with increased odds of MD. Arrythmia, hypertension, diabetes, uncomplicated diabetes, complicated diabetes, cerebrovascular diseases were associated with increased odds of both MD and VD (Table 4). The category of peripheral vascular diseases was associated with increased odds of MD, VD and DLB; the highest OR was found in DLB (OR = 1.51(1.01–2.27)). Complicated diabetes was associated with increased odds of MD, VD and PD; the highest OR was found in PD (OR = 2.51(1.09–5.40)). The category of spinal cord disorders was associated with increased odds of MD, DLB and PD; the highest OR was found in PD (OR = 4.41(2.34–8.30)).

### Comorbidity burden in total sample, according to stage and etiological diagnoses of NCD

The means of the CCI, MWI and HRQOL-CI were 1.8 ± 1.7, 7.3 ± 5.5 and 3.4 ± 2.7, respectively (Table 5). The majority of patients had comorbidity indices higher than 0 (CCI > 0 for n = 2565, 73.9%, MWI > 0 for n = 3295, 95% and HRQOL-CI > 0 for n = 2980, 85.9%).

**Table 5:** Comorbidity burden according to stage and etiological diagnoses of NCD

Comorbidity indices	Total (N = 3470)	Stage of NCD		p-value	Etiological diagnoses of NCD						p-value
		Dementia (n = 2321, 66.9%)	MCI (n = 1149, 33.1%)		AD (n = 1765, 50.9%)	MD (n = 894, 25.8%)	VD (n = 494, 14.2%)	DLB (n = 153, 4.4%)	FTD (n = 83, 2.4%)	PD (n = 81, 2.3%)	
CCI				0.06							< 0.001
Mean ± SD	1.8 ± 1.7	1.8 ± 1.8	1.7 ± 1.7		1.6 ± 1.6	2.1 ± 1.9	2.2 ± 1.9	1.8 ± 1.6	1.6 ± 1.7	1.5 ± 1.6	
Median (Q1-Q3)	1 (0–3)	1 (1–3)	1 (0–3)		1 (0–2)	2 (1–3)	2 (1–3)	2 (1–3)	1 (0–2.5)	1 (0–2)	
Adjusted mean ± SD		1.9 ± 1.8	1.8 ± 1.8		1.6 ± 1.8	2.1 ± 1.7	2.2 ± 1.7	1.9 ± 1.7	1.8 ± 1.7	1.5 ± 1.7	
MWI				< 0.001							< 0.001
Mean ± SD	7.4 ± 5.5	7.7 ± 5.6	6.9 ± 5.1		6.6 ± 5.2	8.3 ± 5.6	8.7 ± 5.5	7.3 ± 5.4	5.7 ± 4.4	6.1 ± 5.8	
Median (Q1-Q3)	6.4 (3.1–10.5)	6.4 (3.1–10.7)	6.0 (3.0–10.0)		5.5 (2.7–9.5)	7.8 (4.0–11.7)	7.8 (4.5–12.4)	5.9 (2.9–11.0)	5.1 (1.6–9.4)	4.3 (1.9–8.3)	
Adjusted mean ± SD		7.6 ± 5.6	7.2 ± 5.6		6.7 ± 5.6	8.2 ± 5.5	8.9 ± 5.3	7.6 ± 5.3	6.8 ± 5.4	6.5 ± 5.3	
HRQOL-CI				< 0.001							< 0.001
Mean ± SD	3.4 ± 2.7	3.5 ± 2.8	3.1 ± 2.6		3.1 ± 2.6	3.7 ± 2.9	3.9 ± 2.8	3.5 ± 2.7	2.8 ± 2.1	3.1 ± 3.0	
Median (Q1-Q3)	3 (1–5)	3 (1–5)	3 (1–5)		3 (1–5)	3 (1–5)	3 (2–6)	3 (2–5)	3 (1–4)	2 (1–4)	
Adjusted mean ± SD		3.5 ± 2.8	3.2 ± 2.8		3.1 ± 2.8	3.6 ± 2.8	3.9 ± 2.7	3.6 ± 2.7	3.3 ± 2.8	3.3 ± 2.7	

*AD* Alzheimer’s disease, *MD* Mixed dementia, *VD* Vascular dementia, *DLB* Dementia with Lewy bodies, *FTD* Frontotemporal dementia, *PD* Parkinson’s disease, *MCI* Mild cognitive impairment, *CCI* Charlson comorbidity index, *MWI* Multimorbidity-weighted index, *HRQOL*-*CI* Health related quality of life comorbidity index

A higher comorbidity burden was found in patients with dementia compared to patients with MCI, as measured by the MWI (7.6 ± 5.6 vs. 6.9 ± 5.1, p < 0.001) and the HRQOL-CI (3.5 ± 2.8 vs. 3.1 ± 2.8, p < 0.001) (Table 5). In contrast, the CCI was not different according to the stage of NCD. Higher comorbidity burden as measured by the MWI and the HRQOL-CI was associated with increased odds of dementia compared to MCI, with OR of 1.02 (1.01–1.03) and OR of 1.03 (1.01–1.06), respectively (Table 4).

Comorbidity burden, as measured by the CCI, the MWI and the HRQOL-CI, was significantly different according to the etiological diagnoses of NCD (Table 5). Higher comorbidity burden, as measured by the CCI and the MWI was associated with increased odds of VD and MD compared to AD. The HRQOL-CI, was associated with increased odds of VD, MD and DLB compared to AD (Table 4).

### Comorbidities and comorbidity burden in AD (reference category) and others NCD

Additional analyses have been performed to compare comorbidity burden between AD and the others considered NCD (MD, VD, DLB, FTD and PD) (supplementary Table 3). Arrythmia, hypertension, peripheral vascular diseases, diabetes, uncomplicated diabetes, complicated diabetes, cerebrovascular diseases, spinal cord disorders and gastric ulcer were more prevalent in the group of others NCD etiologies in comparison to the group with AD. In contrast, a higher prevalence of depression was found in AD patients compared to others NCD patients (30.8% vs. 25.2%). Comorbidity burden as measured by the three comorbidity indices were higher in the group of others NCD etiologies compared to the AD group (supplementary Table 3).

## Discussion

In this study based on a large sample of patients attending a memory center, comorbidity burden as measured by the MWI and the HRQOL-CI was higher in patients with dementia than in patients with MCI. Upon stratifying population according to the etiological diagnoses of NCD, the CCI, the MWI and the HRQOL-CI were higher in VD and mixed dementia than in others considered diagnoses.

Our findings align with previous research reporting a higher comorbidity burden (CIRS-G [8], General Medical Health Rating [30]) in patients with dementia compared to patients with MCI [8, 30]. For etiological diagnoses of NCD, previous studies compared comorbidity burden between AD and only one other NCD etiology [9–11, 31] (a higher mean of CCI in patients with VD, MD and DLB compared to AD). The present study supports these findings through the use of the CCI, MWI and HRQOL-CI. Since comorbidity indices consider a predefined list of comorbidities, examining the distribution of comorbidities by stage and etiology of NCD could provide further explications to our findings.

Compared to the study reported by Chen et al., the prevalence of hypercholesterolemia was higher in our study, but similarly our findings showed a higher prevalence of hypercholesterolemia in patients with MCI than in patients with dementia. Chen et al. explained this difference by the use of statins that reduce the risk of conversion from MCI to dementia. Another possible explanation is that the occurrence of eating disorders related to the progression of cognitive decline could lead to greater reduction of the level of cholesterol in patients with dementia compared to patients with MCI [32]. However, the association between hypercholesterolemia and cognitive decline remains a topic of debate due to various factors, including age, sex, and different cholesterol types (total cholesterol, triglycerides, etc.) [33], which make it difficult to confirm a protective effect of hypercholesterolemia in the present study.

In our study, cerebrovascular diseases were also associated with MCI compared to dementia. Consistent with our findings, a meta-analysis reported that among individuals with post-stroke NCD, two-thirds were at the MCI stage, while one-third had reached the dementia stage [34]. However, these findings may be influenced by factors such as the potential underreporting of comorbidities in dementia compared to MCI, the higher mortality risk related to cerebrovascular diseases, and the fact that MCI patients may eventually progress to dementia (as this is a cross-sectional study). Another potential hypothesis is that cognitive impairment resulting from vascular etiologies, such as cerebrovascular diseases, may be linked to a stable cognitive change [35], which explain why these patients did not progress to dementia stage. Therefore, these findings should be interpreted with caution.

Anemia and arthritis were associated with higher risk of dementia compared to MCI. Both comorbidities were not frequently studied, and previous studies could support our findings: Anemia was reported as a risk factor of dementia [36], as well as a risk factor of progression from MCI to dementia [37]. For arthritis, a higher level of inflammation related to arthritis was also previously found associated with increased cognitive impairment [38].

Regarding etiological diagnoses of NCD, Xiao et al. reported higher prevalence of hypertension, diabetes, congestive heart failure and cerebrovascular diseases in patients with VD than in patients with AD [9], which is similar to our findings. In the present study, a lower prevalence of depression was found in MD compared to AD. Regarding the depression, previous studies have shown inconsistent findings, making challenging to interpret: some studies have found higher depression in patients with AD compared to patients with VD [31], and higher depression in patients with DLB compared to patients with AD [39], while one meta-analysis indicated a similar prevalence of depression in patients with FTD, AD and VD [40]. Nevertheless, the meta-analysis did not consider mixed dementia which precludes the comparison with our findings.

MD has rarely been considered in studies on comorbidity distribution by NCD etiology, limiting comparisons with our findings. Two studies found higher prevalence of hypertension and cerebrovascular diseases in MD than in AD patients [10, 41], similarly to our findings. In our study peripheral vascular diseases were also, more prevalent in MD than in AD patients. According to the vascular theory, hypertension, peripheral vascular diseases and diabetes may contribute to neurovascular dysfunction in patients with AD [42], which may lead to MD. As consequence, comorbidity profile (both prevalence of comorbidities and comorbidity burden) of MD was not similar to comorbidity profile of AD, but there was some resemblance between patients with MD and VD regarding their comorbidity profile.

A study by Nagar et al. conducted in US and including 551 individuals with dementia or AD [43] reported a hypertension’s prevalence of 81.85% which was higher than our findings (50.2%). In the study by Nagar et al., hypertension was defined by grouping multiple potential diagnoses, whereas in our study, we relied on physician-documented medical diagnoses from clinical records. This methodological difference may account for the discrepancy in findings.

Our study extends findings of a previous study by Artaz et al. conducted in France and including 579 patients with AD (age: 77.4 ± 7.1; percentage of men: 28%) from 19 memory centers [44]. Similar to our findings, the prevalence was found to be 45% (vs. 46.2% in our study) for hypertension and 24% (vs. 21.4% in our study) for hypercholesterolemia. In the study by Artaz et al. the prevalence of diabetes was 10% (vs. 16.7% in our study) and it was higher in men than women (15% vs. 8%). The AD patients in our study were older with a higher proportion of men compared to study reported by Artaz et al., which may explain the higher prevalence of diabetes reported by our study.

This study has several strengths such as the large sample size, which helped us to consider both stage and etiological diagnoses of NCD. To the best of our knowledge this is the first study that involved six different etiological diagnoses of NCD, in order to describe comorbidities. Moreover, this study was conducted using data from a memory center in a real-life context, suggesting that our findings may be generalizable to patients in similar setting. Three different comorbidity indices were calculated in this study allowing to consolidate our findings and take into account the differences in calculation between the indices. This study focused on an extensive list of comorbidities.

Several limitations should be considered when interpreting our findings. In this study, comorbidities were reported based on physician-confirmed medical records. Since multiple physicians collected this data through open-ended questions, measurement bias and underestimation may potentially affect prevalence estimation. Comparison of our findings with those reported by previous studies, did not show a higher discrepancy, indicating that these biases were limited. Moreover, these biases seem to be consistent across stage/etiologies of NCD, allowing us to understand comorbidities’ distribution. Despite the large sample size, certain diagnoses such as PD and FTD were less represented than others NCD diagnoses, which may affect the comparison between etiologies. The diagnoses of NCD was mainly based on a clinico-radiological framework, and the majority of cases lacked pathophysiological biomarkers, especially for the diagnosis of AD. This could be associated with a higher likelihood of diagnostic errors, even if the study was conducted in a specialized memory clinic with trained physicians. Additionally, the overlap between NCD etiologies was likely underestimated due to the absence of neuropathological assessments, which may have led to an underestimation of brain copathologies, even when considering"mixed dementia"as an etiology. The pharmaceutical treatments of comorbidities were not taken into account in this study, which may impact the comparison between patients. Finally, this study was cross-sectional which does not allow to establish causality between comorbidity burden and stage/etiological diagnoses of NCD.

In conclusion, this study highlighted that comorbidities’ prevalence as well as comorbidity burden as measured by the CCI, the MWI and the HRQOL-CI varied according to both the stages and the etiological diagnoses of NCD. The identification of different comorbidity profiles depending of NCD stages and various etiological diagnoses may contribute to more tailored management and interventions for patients with NCD. Future studies should consider the stage and the etiological diagnoses, when studying the effect of the comorbidity burden on the progression of NCD.

## Supplementary Information

Below is the link to the electronic supplementary material.

Supplementary file1 (DOCX 28 KB)

## Data Availability

The data are not publicly available due to regulations and ethical restrictions.

## References

[1] Health TLP (2021) Reinvigorating the public health response to dementia. Lancet Public Health 6:e696. 10.1016/S2468-2667(21)00215-2 34563278 10.1016/S2468-2667(21)00215-2 PMC8516159

[2] Cao Q, Tan C-C, Xu W, Hu H, Cao X-P, Dong Q, Tan L, Yu J-T (2020) The prevalence of dementia: a systematic review and meta-analysis. J Alzheimers Dis 73:1157–1166. 10.3233/JAD-191092 31884487 10.3233/JAD-191092

[3] Livingston G, Huntley J, Sommerlad A, Ames D, Ballard C, Banerjee S, Brayne C, Burns A, Cohen-Mansfield J, Cooper C, Costafreda SG, Dias A, Fox N, Gitlin LN, Howard R, Kales HC, Kivimäki M, Larson EB, Ogunniyi A, Orgeta V, Ritchie K, Rockwood K, Sampson EL, Samus Q, Schneider LS, Selbæk G, Teri L, Mukadam N (2020) Dementia prevention, intervention, and care: 2020 report of the Lancet Commission. Lancet 396:413–446. 10.1016/S0140-6736(20)30367-6 32738937 10.1016/S0140-6736(20)30367-6 PMC7392084

[4] Haaksma ML, Vilela LR, Marengoni A, Calderón-Larrañaga A, Leoutsakos J-MS, Rikkert MGMO, Melis RJF (2017) Comorbidity and progression of late onset Alzheimer’s disease: a systematic review. PLoS One 12:e0177044. 10.1371/journal.pone.0177044 28472200 10.1371/journal.pone.0177044 PMC5417646

[5] Temedda MN, Garnier-Crussard A, Mouchoux C, Dauphinot V (2024) Association between comorbidity indices and functional autonomy in individuals with cognitive impairment: a systematic review. J Prev Alzheimers Dis. 10.14283/jpad.2024.51 39044516 10.14283/jpad.2024.51 PMC12275834

[6] Bertsias A, Symvoulakis E, Tziraki C, Panagiotakis S, Mathioudakis L, Zaganas I, Basta M, Boumpas D, Simos P, Vgontzas A, Lionis C (2020) Cognitive impairment and dementia in primary care: current knowledge and future directions based on findings from a large cross-sectional study in Crete, Greece. Front Med 7:592924. 10.3389/fmed.2020.592924 10.3389/fmed.2020.592924 PMC7719838 33330553

[7] Chen T-B, Yiao S-Y, Sun Y, Lee H-J, Yang S-C, Chiu M-J, Chen T-F, Lin K-N, Tang L-Y, Lin C-C, Wang P-N (2017) Comorbidity and dementia: a nationwide survey in Taiwan. PLoS One 12:e0175475. 10.1371/journal.pone.0175475 28403222 10.1371/journal.pone.0175475 PMC5389824

[8] Liao W, Hamel REG, Olde Rikkert MGM, Oosterveld SM, Aalten P, Verhey FRJ, Scheltens P, Sistermans N, Pijnenburg YAL, van der Flier WM, Ramakers IHGB, Melis RJF (2016) A profile of the clinical course of cognition and comorbidity in mild cognitive impairment and dementia study (the 4C study): two complementary longitudinal, clinical cohorts in the Netherlands. BMC Neurol 16:242. 10.1186/s12883-016-0750-9 27884130 10.1186/s12883-016-0750-9 PMC5123233

[9] Xiao X, Xiang S, Xu Q, Li J, Xiao J, Si Y (2023) Comorbidity among inpatients with dementia: a preliminary cross-sectional study in west China. Aging Clin Exp Res 35:659–667. 10.1007/s40520-023-02349-3 36754914 10.1007/s40520-023-02349-3 PMC9908504

[10] Zekry D, Herrmann FR, Grandjean R, Meynet M-P, Michel J-P, Gold G, Krause K-H (2008) Demented versus non-demented very old inpatients: the same comorbidities but poorer functional and nutritional status. Age Ageing 37:83–89. 10.1093/ageing/afm132 17971391 10.1093/ageing/afm132

[11] Fereshtehnejad S-M, Damangir S, Cermakova P, Aarsland D, Eriksdotter M, Religa D (2014) Comorbidity profile in dementia with Lewy bodies versus Alzheimer’s disease: a linkage study between the Swedish dementia registry and the Swedish National Patient Registry. Alzheimers Res Ther 6:65. 10.1186/s13195-014-0065-2 25478027 10.1186/s13195-014-0065-2 PMC4255539

[12] de Groot V, Beckerman H, Lankhorst GJ, Bouter LM (2003) How to measure comorbidity. a critical review of available methods. J Clin Epidemiol 56:221–229. 10.1016/s0895-4356(02)00585-1 12725876 10.1016/s0895-4356(02)00585-1

[13] Wei MY, Ratz D, Mukamal KJ (2020) Multimorbidity in Medicare beneficiaries: performance of an ICD-coded multimorbidity-weighted index. J Am Geriatr Soc 68:999–1006. 10.1111/jgs.16310 31917465 10.1111/jgs.16310 PMC7234913

[14] Temedda MN, Garnier-Crussard A, Moutet C, Mouchoux C, Dauphinot V (2025) Association between comorbidity indices and nursing home admission in patients with Alzheimer’s disease: a longitudinal observational study using the MEMORA cohort. BMC Geriatr 25:254. 10.1186/s12877-025-05898-6 40240995 10.1186/s12877-025-05898-6 PMC12004650

[15] Wei MY, Kabeto MU, Langa KM, Mukamal KJ (2018) Multimorbidity and physical and cognitive function: performance of a new multimorbidity-weighted index. J Gerontol A Biol Sci Med Sci 73:225–232. 10.1093/gerona/glx114 28605457 10.1093/gerona/glx114 PMC5861895

[16] Dauphinot V, Moutet C, Rouch I, Verdurand M, Mouchoux C, Delphin-Combe F, Gaujard S, Krolak-Salmon P, MEMORA group (2019) A multicenter cohort study to investigate the factors associated with functional autonomy change in patients with cognitive complaint or neurocognitive disorders: the MEMORA study protocol. BMC Geriatr 19:191. 10.1186/s12877-019-1204-1 31319809 10.1186/s12877-019-1204-1 PMC6637582

[17] McKhann GM, Knopman DS, Chertkow H, Hyman BT, Jack CR, Kawas CH, Klunk WE, Koroshetz WJ, Manly JJ, Mayeux R, Mohs RC, Morris JC, Rossor MN, Scheltens P, Carrillo MC, Thies B, Weintraub S, Phelps CH (2011) The diagnosis of dementia due to Alzheimer’s disease: recommendations from the National Institute on Aging-Alzheimer’s Association workgroups on diagnostic guidelines for Alzheimer’s disease. Alzheimers Dement 7:263–269. 10.1016/j.jalz.2011.03.005 21514250 10.1016/j.jalz.2011.03.005 PMC3312024

[18] Sachdev P, Kalaria R, O’Brien J, Skoog I, Alladi S, Black SE, Blacker D, Blazer D, Chen C, Chui H, Ganguli M, Jellinger K, Jeste DV, Pasquier F, Paulsen J, Prins N, Rockwood K, Roman G, Scheltens P (2014) Diagnostic criteria for vascular cognitive disorders: a VASCOG statement. Alzheimer Dis Assoc Disord 28:206–218. 10.1097/WAD.0000000000000034 24632990 10.1097/WAD.0000000000000034 PMC4139434

[19] McKeith IG, Boeve BF, Dickson DW, Halliday G, Taylor J-P, Weintraub D, Aarsland D, Galvin J, Attems J, Ballard CG, Bayston A, Beach TG, Blanc F, Bohnen N, Bonanni L, Bras J, Brundin P, Burn D, Chen-Plotkin A, Duda JE, El-Agnaf O, Feldman H, Ferman TJ, Ffytche D, Fujishiro H, Galasko D, Goldman JG, Gomperts SN, Graff-Radford NR, Honig LS, Iranzo A, Kantarci K, Kaufer D, Kukull W, Lee VMY, Leverenz JB, Lewis S, Lippa C, Lunde A, Masellis M, Masliah E, McLean P, Mollenhauer B, Montine TJ, Moreno E, Mori E, Murray M, O’Brien JT, Orimo S, Postuma RB, Ramaswamy S, Ross OA, Salmon DP, Singleton A, Taylor A, Thomas A, Tiraboschi P, Toledo JB, Trojanowski JQ, Tsuang D, Walker Z, Yamada M, Kosaka K (2017) Diagnosis and management of dementia with Lewy bodies: Fourth consensus report of the DLB Consortium. Neurology 89:88–100. 10.1212/WNL.0000000000004058 28592453 10.1212/WNL.0000000000004058 PMC5496518

[20] Rascovsky K, Hodges JR, Knopman D, Mendez MF, Kramer JH, Neuhaus J, van Swieten JC, Seelaar H, Dopper EGP, Onyike CU, Hillis AE, Josephs KA, Boeve BF, Kertesz A, Seeley WW, Rankin KP, Johnson JK, Gorno-Tempini M-L, Rosen H, Prioleau-Latham CE, Lee A, Kipps CM, Lillo P, Piguet O, Rohrer JD, Rossor MN, Warren JD, Fox NC, Galasko D, Salmon DP, Black SE, Mesulam M, Weintraub S, Dickerson BC, Diehl-Schmid J, Pasquier F, Deramecourt V, Lebert F, Pijnenburg Y, Chow TW, Manes F, Grafman J, Cappa SF, Freedman M, Grossman M, Miller BL (2011) Sensitivity of revised diagnostic criteria for the behavioural variant of frontotemporal dementia. Brain 134:2456–2477. 10.1093/brain/awr179 21810890 10.1093/brain/awr179 PMC3170532

[21] Postuma RB, Berg D, Stern M, Poewe W, Olanow CW, Oertel W, Obeso J, Marek K, Litvan I, Lang AE, Halliday G, Goetz CG, Gasser T, Dubois B, Chan P, Bloem BR, Adler CH, Deuschl G (2015) MDS clinical diagnostic criteria for Parkinson’s disease. Mov Disord 30:1591–1601. 10.1002/mds.26424 26474316 10.1002/mds.26424

[22] Regier DA, Kuhl EA, Kupfer DJ (2013) The DSM-5: classification and criteria changes. World Psychiatry 12:92–98. 10.1002/wps.20050 23737408 10.1002/wps.20050 PMC3683251

[23] Albert MS, DeKosky ST, Dickson D, Dubois B, Feldman HH, Fox NC, Gamst A, Holtzman DM, Jagust WJ, Petersen RC, Snyder PJ, Carrillo MC, Thies B, Phelps CH (2011) The diagnosis of mild cognitive impairment due to Alzheimer’s disease: recommendations from the National Institute on Aging-Alzheimer’s Association workgroups on diagnostic guidelines for Alzheimer’s disease. Alzheimers Dement 7:270–279. 10.1016/j.jalz.2011.03.008 21514249 10.1016/j.jalz.2011.03.008 PMC3312027

[24] Charlson ME, Pompei P, Ales KL, MacKenzie CR (1987) A new method of classifying prognostic comorbidity in longitudinal studies: development and validation. J Chronic Dis 40:373–383. 10.1016/0021-9681(87)90171-8 3558716 10.1016/0021-9681(87)90171-8

[25] Wei MY, Kawachi I, Okereke OI, Mukamal KJ (2016) Diverse cumulative impact of chronic diseases on physical health-related quality of life: implications for a measure of multimorbidity. Am J Epidemiol 184:357–365. 10.1093/aje/kwv456 27530335 10.1093/aje/kwv456 PMC5013885

[26] Mukherjee B, Ou H-T, Wang F, Erickson SR (2011) A new comorbidity index: the health-related quality of life comorbidity index. J Clin Epidemiol 64:309–319. 10.1016/j.jclinepi.2010.01.025 21147517 10.1016/j.jclinepi.2010.01.025

[27] Hespanhol L, Vallio CS, Costa LM, Saragiotto BT (2019) Understanding and interpreting confidence and credible intervals around effect estimates. Braz J Phys Ther 23:290. 10.1016/j.bjpt.2018.12.006 30638956 10.1016/j.bjpt.2018.12.006 PMC6630113

[28] Ahluwalia SC, Gross CP, Chaudhry SI, Leo-Summers L, Van Ness PH, Fried TR (2011) Change in comorbidity prevalence with advancing age among persons with heart failure. J Gen Intern Med 26:1145–1151. 10.1007/s11606-011-1725-6 21573881 10.1007/s11606-011-1725-6 PMC3181289

[29] Zhang Y, Natale G, Clouston S (2021) Incidence of mild cognitive impairment, conversion to probable dementia, and mortality. Am J Alzheimers Dis Other Demen 36:15333175211012236. 10.1177/15333175211012235 34032119 10.1177/15333175211012235 PMC8715729

[30] Lyketsos CG, Toone L, Tschanz J, Rabins PV, Steinberg M, Onyike CU, Corcoran C, Norton M, Zandi P, Breitner JCS, Welsh-Bohmer K (2005) Population-based study of medical comorbidity in early dementia and “cognitive impairment, no dementia (CIND)”: association with functional and cognitive impairment: the cache county study. Am J Geriatr Psychiatry 13:656–664. 10.1097/00019442-200508000-00004 16085781 10.1176/appi.ajgp.13.8.656

[31] Wang Q-H, Wang X, Bu X-L, Lian Y, Xiang Y, Luo H-B, Zou H-Q, Pu J, Zhou Z-H, Cui X-P, Wang Q-S, Shi X-Q, Han W, Wu Q, Chen H-S, Lin H, Gao C-Y, Zhang L-L, Xu Z-Q, Zhang M, Zhou H-D, Wang Y-J (2017) Comorbidity burden of dementia: a hospital-based retrospective study from 2003 to 2012 in seven cities in China. Neurosci Bull 33:703–710. 10.1007/s12264-017-0193-3 29134450 10.1007/s12264-017-0193-3 PMC5725390

[32] Slaughter SE, Hayduk LA (2012) Contributions of environment, comorbidity, and stage of dementia to the onset of walking and eating disability in long-term care residents. J Am Geriatr Soc 60:1624–1631. 10.1111/j.1532-5415.2012.04116.x 22985138 10.1111/j.1532-5415.2012.04116.x

[33] Boccardi V, Mancinetti F, Guazzarini AG, Murasecco I, Melis F, Bastiani P, Scamosci M, Cecchetti R, Mecocci P (2025) Sex-specific associations between serum lipid levels and cognitive performance in older adults: results from a cross-sectional real-world study. Aging Clin Exp Res 37:62. 10.1007/s40520-025-02976-y 40021569 10.1007/s40520-025-02976-y PMC11870876

[34] Barbay M, Diouf M, Roussel M, Godefroy O, GRECOGVASC study group (2018) Systematic review and meta-analysis of prevalence in post-stroke neurocognitive disorders in hospital-based studies. Dement Geriatr Cogn Disord 46:322–334. 10.1159/000492920 30504699 10.1159/000492920

[35] Stephan BC, Matthews FE, Khaw K-T, Dufouil C, Brayne C (2009) Beyond mild cognitive impairment: vascular cognitive impairment, no dementia (VCIND). Alzheimers Res Ther 1:4. 10.1186/alzrt4 19674437 10.1186/alzrt4 PMC2719105

[36] Qiang Y-X, Deng Y-T, Zhang Y-R, Wang H-F, Zhang W, Dong Q, Feng J-F, Cheng W, Yu J-T (2023) Associations of blood cell indices and anemia with risk of incident dementia: a prospective cohort study of 313,448 participants. Alzheimers Dement 19:3965–3976. 10.1002/alz.13088 37102212 10.1002/alz.13088

[37] Stephan BCM, Brayne C, Savva GM, Matthews FE (2011) Occurrence of medical co-morbidity in mild cognitive impairment: implications for generalisation of MCI research. Age Ageing 40:501–507. 10.1093/ageing/afr057 21673136 10.1093/ageing/afr057 PMC3290328

[38] Sangha PS, Thakur M, Akhtar Z, Ramani S, Gyamfi RS (2020) The link between rheumatoid arthritis and dementia: a review. Cureus 12:e7855. 10.7759/cureus.7855 32489719 10.7759/cureus.7855 PMC7255531

[39] Chiu P-Y, Wang C-W, Tsai C-T, Li S-H, Lin C-L, Lai T-J (2017) Depression in dementia with Lewy bodies: a comparison with Alzheimer’s disease. PLoS One 12:e0179399. 10.1371/journal.pone.0179399 28617831 10.1371/journal.pone.0179399 PMC5472293

[40] Chakrabarty T, Sepehry AA, Jacova C, Hsiung GY (2015) The prevalence of depressive symptoms in frontotemporal dementia: a meta-analysis. Dement Geriatr Cogn Disord. 10.1159/000369882 25662033 10.1159/000369882

[41] Moreno Cervantes C, Mimenza Alvarado A, Aguilar Navarro S, Alvarado Ávila P, Gutiérrez Gutiérrez L, Juárez Arellano S, Ávila Funes JA (2017) Factores asociados a la demencia mixta en comparación con demencia tipo Alzheimer en adultos mayores mexicanos. Neurologia 32:309–315. 10.1016/j.nrl.2015.12.006 26971058 10.1016/j.nrl.2015.12.006

[42] Custodio N, Montesinos R, Lira D, Herrera-Pérez E, Bardales Y, Valeriano-Lorenzo L (2017) Mixed dementia: a review of the evidence. Dement Neuropsychol 11:364–370. 10.1590/1980-57642016dn11-040005 29354216 10.1590/1980-57642016dn11-040005 PMC5769994

[43] Nagar SD, Pemu P, Qian J, Boerwinkle E, Cicek M, Clark CR, Cohn E, Gebo K, Loperena R, Mayo K, Mockrin S, Ohno-Machado L, Ramirez AH, Schully S, Able A, Green A, Zuchner S, Jordan IK, Meller R (2022) Investigation of hypertension and type 2 diabetes as risk factors for dementia in the All of Us cohort. Sci Rep 12:19797. 10.1038/s41598-022-23353-z 36396674 10.1038/s41598-022-23353-z PMC9672061

[44] Artaz M-A, Boddaert J, Hériche-Taillandier E, Dieudonné B, Verny M (2006) le groupe REAL.FR, [Medical comorbidity in Alzheimer’s disease: baseline characteristics of the REAL.FR Cohort]. Rev Med Interne 27:91–97. 10.1016/j.revmed.2005.10.007 16359758 10.1016/j.revmed.2005.10.007

