# Supplementary material for: Comorbidities and comorbidity burden in patients with neurocognitive disorders: findings from the MEMORA Cohort Study
Source: Eur Geriatr Med. 2025 Aug 27;16(6):2153–67. doi: 10.1007/s41999-025-01288-8 (PMC12743666; doi:10.1007/s41999-025-01288-8)
Supplement: Supplementary file 1 — Supplementary file1 (DOCX 28 KB) [file 41999_2025_1288_MOESM1_ESM.docx]

**Supplementary tables**

**Supplementary table 1: Comorbidity indices**

| Comorbidity indices | Comorbidities | Weights |
| --- | --- | --- |
| CCI | Congestive heart failure; Myocardial infraction; Peripheral vascular diseases; Cerebrovascular diseases; Uncomplicated diabetes; Renal diseases; Chronic obstructive pulmonary diseases; Mild liver diseases  Connective tissue diseases; Ulcer gastric | 1 |
|  | Complicated diabetes; Hemiplegia; Non metastatic cancer | 2 |
|  | Moderate to severe liver diseases | 3 |
|  | Metastatic solid tumor; Acquired immune deficiency syndrome | 6 |
| MWI | Congestive heart failure  Arrhythmia  Valvular diseases  Angina  Myocardial infraction  Hypertension  Peripheral vascular diseases  Anemia  Diabetes  Hypercholesterolemia  Stroke  Transient ischemic attack  Depression  Renal diseases  Chronic obstructive pulmonary diseases  Asthma  Total cancer  Liver diseases  Osteoarthritis  Osteoporosis  Arthritis  Hip replacement  Knee replacement  Herniated disc  Acquired immune deficiency syndrome  Ulcer gastric  Glaucoma  Cataract | 4.77  1.33  0.416  2.2  1.73  1.53  3.25  1.82  2.67  0.343  3.79  1.24  1.29  3.98  4.32  1.62  1.61  0.293  3.52  0.997  3.79  3.55  9.11  3.27  2.91  1.08  0.427  0.288 |
| HRQOL-CI | Anxiety/depression; Vision disorders; Thyroid disorders; Esophageal disorders; Arrhythmia; Asthma; Hypertension; Gastric ulcer | 1 |
|  | Chronic obstructive pulmonary diseases; Spinal cord disorders; Peripheral vascular disorders; Diabetes; Hepatitis; Osteoarthritis; Ischemic heart diseases; Systemic lupus erythematosus | 2 |
|  | Congestive heart failure; Arthritis, Paralysis/ Hemiplegia | 3 |
| CCI: Charlson comorbidity index; MWI: Multimorbidity-weighted index; HRQOL-CI: Health related quality of life comorbidity index | | |

**Supplementary table 2: Adjusted prevalence of comorbidities according to the stage and etiological diagnoses of NCD**

| Comorbidities | Stage of NCD | | p-value | Etiological diagnoses of NCD | | | | | | p-value | |
| --- | --- | --- | --- | --- | --- | --- | --- | --- | --- | --- | --- |
|  | Dementia  (n = 2321, 66.9%) | MCI  (n = 1149, 33.1%) |  | AD  (n = 1765, 50.9%) | MD  (n = 894, 25.8%) | VD  (n = 494, 14.2%) | DLB  (n = 153, 4.4%) | FTD  (n = 83, 2.4%) | PD  (n = 81, 2.3%) |  |  |
|  | % (95%CI) | % (95%CI) |  | % (95%CI) | % (95%CI) | % (95%CI) | % (95%CI) | % (95%CI) | % (95%CI) |  |  |
| CHF | 18.3 (16.8-20.0) | 16.7 (14.6-18.9) | <0.001 | 17.3 (15.6-19.2) | 19.3 (16.8-22.0) | 17.9 (14.8-21.6) | 17.0 (11.9-23.8) | 12.3 (6.8-21.0) | 17.0 (10.3-26.6) | <0.001 | |
| Arrhythmia |  |  |  | 16.8 (15.1-18.6) | 18.0 (15.6-206) | 16.9 (13.8-20.4) | 16.3 (11.3-22.9) | 13.1 (7.4-22.0) | 16.5 (9.9-26.0) | <0.001 | |
| Valvular diseases |  |  |  | 3.8 (3.0-4.8) | 3.9 (2.8-5.4) | 3.6 (2.3-5.6) | 3.4 (1.5-7.6) | 0 | 3.6 (1.2-10.1) | <0.001 | |
| Hypertension | 51.0 (49.0-53.1) | 48.5 (45.6-51.4) | <0.001 | 50.4 (48.1-52.7) | 51.8 (48.5-55.0) | 49.2 (44.8-53.6) | 48.7 (40.9-56.5) | 42.8 (32.7-53.5) | 45.3 (34.9-56.1) | <0.001 | |
| Hypercholesterolemia | 22.6 (21.0-24.4) | 22.5 (20.2-25.0) | 0.20 | 22.8 (20.9-24.8) | 22.4 (19.8-25.2) | 22.5 (19.0-26.4) | 22.3 (16.4-29.5) | 23.3 (15.5-33.4) | 21.7 (14.1-31.8) | <0.001 | |
| PVD |  |  |  | 19.1 (17.3-21.0) | 19.8 (17.4-22.6) | 19.0 (15.8-22.7) | 18.4 (13.0-25.3) | 16.2 (9.8-25.6) | 18.5 (11.6-28.3) | <0.001 | |
| Anemia | 6.9 (6.0-8.0) | 6.4 (5.1-8.0) | <0.001 |  |  |  |  |  |  |  | |
| Diabetes | 21.2 (19.6-22.9) | 19.5 (17.3-21.9) | <0.001 | 20.0 (18.2-22.0) | 20.2 (17.7-23.0) | 22.4 (19.0-26.3) | 22.6 (16.7-29.9) | 23.6 (15.8-33.8) | 20.4 (13.0-30.4) | <0.001 | |
| Uncomplicated diabetes |  |  |  | 14.4 (12.9-16.2) | 14.5 (12.4-17.0) | 16.4 (13.4-19.9) | 16.5 (11.5-23.2) | 17.7 (11.0-27.3) | 15.1 (8.9-24.5) | <0.001 | |
| Complicated diabetes |  |  |  | 5.6 (4.6-6.8) | 5.7 (4.3-7.4) | 6.1 (4.3-8.5) | 6.1 (3.3-11.0) | 6.0 (2.6-13.3) | 5.2 (2.1-12.3) | <0.001 | |
| CVD | 13.8 (12.4-15.2) | 13.8 (11.9-15.9) | 0.99 | 13.5 (12.0-15.2) | 14.0 (11.8-16.4) | 14.1 (11.3-17.5) | 14.1 (9.5-20.5) | 13.6 (7.8-22.6) | 14.7 (8.6-24.0) | <0.001 | |
| Spinal cord disorders |  |  |  | 6.8 (5.7-8.0) | 7.1 (5.6-8.9) | 6.7 (4.8-9.2) | 6.7 (3.7-11.9) | 5.7 (2.4-13.0) | 6.4 (2.8-14.0) | <0.001 | |
| Depression |  |  |  | 18.6 (26.5-30.7) | 27.2 (24.4-30.2) | 27.4 (23.7-31.5) | 27.7 (21.2-35.2) | 30.8 (21.9-41.4) | 27.6 (19.1-38.2) | <0.001 | |
| Arthritis | 4.8 (4.0-5.8) | 4.9 (3.8-6.3) | 0.51 |  |  |  |  |  |  |  | |
| Vision disorders |  |  |  | 22.3 (20.4-24.3) | 23.4 (20.7-26.3) | 21.1 (17.7-24.9) | 20.2 (14.6-27.3) | 15.8 (9.5-25.1) | 19.6 (12.4-29.5) | <0.001 | |
| CHF: Congestive heart failure; PVD: Peripheral vascular diseases; CVD: Cerebrovascular diseases; AD: Alzheimer’s disease; MD: Mixed dementia; VD: Vascular dementia; DLB: Dementia with Lewy bodies; FTD: Frontotemporal dementia; PD: Parkinson’s disease; CI: Confidence interval; | | | | | | | | | | |  |

**Supplementary table 3: prevalence of comorbidities in patients with AD and others NCD**†

| Comorbidities/comorbidity burden | Etiological diagnoses of NCD | | | | | Others NCD† vs. AD (ref) | |
| --- | --- | --- | --- | --- | --- | --- | --- |
|  | AD  (n = 1765, 50.9%) | | Others NCD†  (n = 1705, 49.1%) | | p-value |  |  |
|  | n | % (95%CI ) | n | % (95%CI ) |  | Adjusted OR (95%IC) | p-value |
| Myocardial |  |  |  |  |  |  |  |
| CHF | 289 | 16.4 (14.7-18.2) | 328 | 19.2 (17.4-21.2) | 0.031 | 1.14 (0.96-1.37) | 0.14 |
| Arrhythmia | 261 | 14.8 (13.2-16.5) | 328 | 19.2 (17.4-21.2) | <0.001 | 1.34 (1.12-1.60) | 0.002 |
| Valvular diseases | 63 | 3.6 (2.8-4.5) | 67 | 3.9 (3.1-5.0) | 0.64 |  |  |
| Angina | 17 | 1.0 (0.6-1.5) | 22 | 1.3 (.9-1.9) | 0.45 |  |  |
| MI | 48 | 2.7 (2.1-3.6) | 60 | 3.5 (2.7-4.5) | 0.21 |  |  |
| Ischemic heart diseases | 82 | 4.6 (3.8-5.7) | 97 | 5.7 (4.7-6.9) | 0.19 |  |  |
| Vascular |  |  |  |  |  |  |  |
| Hypertension | 816 | 46.2 (43.9-48.6) | 926 | 54.3 (51.9-56.7) | <0.001 | 1.42 (1.24-1.63) | <0.001 |
| Hypercholesterolemia | 378 | 21.4 (19.6-23.4) | 406 | 23.8 (21.9-25.9) | 0.10 |  |  |
| PVD | 293 | 16.6 (14.9-18.4) | 371 | 21.8 (19.9-23.8) | <0.001 | 1.39 (1.17-1.65) | <0.001 |
| Anemia | 105 | 05.9 (04.9-07.2) | 129 | 7.6 (6.4-8.9) | 0.07 |  |  |
| Endocrine |  |  |  |  |  |  |  |
| Diabetes | 294 | 16.7 (15.0-18.5) | 422 | 24.8 (22.8-26.9) | <0.001 | 1.57 (1.33-1.87) | <0.001 |
| Uncomplicated diabetes | 223 | 12.6 (11.2-14.3) | 295 | 17.3 (15.6-19.2) | <0.001 | 1.36 (1.12-1.65) | <0.001 |
| Complicated diabetes | 71 | 4.0 (3.2-5.0) | 127 | 7.4 (6.3-8.8) | <0.001 | 1.88 (1.39-2.55) | <0.001 |
| Thyroid disorders | 233 | 13.2 (11.7-14.9) | 189 | 11.1 (9.7-12.7) | 0.06 |  |  |
| Neurological |  |  |  |  |  |  |  |
| CVD | 114 | 6.5 (5.4-7.7) | 364 | 21.3 (19.5-23.4) | <0.001 | 3.84 (3.08-4.82) | <0.001 |
| Hemiplegia | 14 | 0.8 (0.5-1.3) | 21 | 1.2 (0.8-1.9) | 0.26 |  |  |
| Spinal cord disorders | 90 | 5.1 (4.2-6.2) | 146 | 8.6 (7.3-10.0) | <0.001 | 1.74 (1.33-2.30) | <0.001 |
| Psychiatric |  |  |  |  |  |  |  |
| Depression | 543 | 30.8 (28.7-33.0) | 430 | 25.2 (23.2-27.3) | <0.001 | 0.80 (0.68-0.93) | 0.003 |
| Renal |  |  |  |  |  |  |  |
| Chronic renal diseases | 234 | 13.3 (11.8-14.9) | 250 | 14.7 (13.1-16.4) | 0.25 |  |  |
| Pulmonary |  |  |  |  |  |  |  |
| COPD/Asthma | 367 | 20.8 (19.0-22.7) | 387 | 22.7 (20.8-24.7) | 0.19 |  |  |
| Cancer |  |  |  |  |  |  |  |
| Total Cancer | 328 | 18.6 (16.8-20.5) | 346 | 20.3 (18.5-22.3) | 0.22 |  |  |
| Non-Metastatic | 312 | 17.7 (16.0-19.5) | 328 | 19.2 (17.4-21.2) | 0.25 |  |  |
| Metastatic solid tumor | 16 | 0.9 (0.6-1.5) | 18 | 1.1 (0.7-1.7) | 0.78 |  |  |
| Liver |  |  |  |  |  |  |  |
| Mild | 33 | 1.9 (1.3-2.6) | 38 | 2.2 (1.6-3.0) | 0.53 |  |  |
| Moderate to severe | 17 | 1.0 (0.6-1.5) | 26 | 1.5 (1.0-2.2) | 0.18 |  |  |
| Hepatitis | 28 | 1.6 (1.1-2.3) | 36 | 2.1 (1.5-2.9) | 0.31 |  |  |
| Connective |  |  |  |  |  |  |  |
| Arthritis | 79 | 4.5 (3.6-5.5) | 89 | 5.2 (04.3-06.4) | 0.35 |  |  |
| Osteoporosis | 172 | 9.7 (8.4-11.2) | 144 | 8.4 (7.2-9.9) | 0.20 |  |  |
| Osteoarthritis | 272 | 15.4 (13.8-17.2) | 276 | 16.2 (14.5-18.0) | 0.56 |  |  |
| Hip replacement | 119 | 6.8 (5.7-8.1) | 123 | 7.2 (6.1-8.5) | 0.93 |  |  |
| Knee replacement | 53 | 3.0 (2.3-3.9) | 53 | 3.1 (2.4-4.0) | 0.63 |  |  |
| Ophthalmologic |  |  |  |  |  |  |  |
| Vision disorders | 370 | 21.0 (19.1-22.9) | 396 | 23.2 (21.3-25.3) | 0.12 |  |  |
| Infections/Immune |  |  |  |  |  |  |  |
| AIDS | 2 | 0.1 (0.0-0.4) | 2 | 0.1 (0.0-0.4) | 1* |  |  |
| Digestives |  |  |  |  |  |  |  |
| Gastric ulcer | 103 | 5.8 (4.8-7.0) | 137 | 8.0 (6.8-9.4) | 0.012 | 1.40 (1.08-1.84) | 0.012 |
| Esophageal disorders | 19 | 1.1 (0.7-1.7) | 19 | 1.1 (0.7-1.7) | 1 |  |  |
| Comorbidity indices, mean ± SD | | | | | | | |
| CCI | 1.6 ± 1.6 | | 2.04 ± 1.8 | | <0.001 | 1.17 (1.12-1.22) | <0.001 |
| MWI | 6.6 ± 5.2 | | 8.2 ± 5.6 | | <0.001 | 1.05 (1.04-1.07) | <0.001 |
| HRQOL-CI | 3.1 ± 2.6 | | 3.6 ± 2.8 | | <0.001 | 1.08 (1.05-1.10) | <0.001 |
| NCD: Neurocognitive disorders; CHF: Congestive heart failure; MI: Myocardial infraction; PVD: Peripheral vascular diseases; COPD: Chronic obstructive pulmonary diseases; CVD: Cerebrovascular diseases; AIDS: Acquired immune deficiency syndrome; MCI: Mild cognitive impairment; CI: Confidence interval; p: p-value, *: Fisher's exact test, † others NCD included mixed dementia, vascular dementia, dementia with Lewy bodies, frontotemporal dementia and Parkinson’s disease; CCI : Charlson comorbidity index; MWI: Multimorbidity-weighted index; HRQOL-CI: Health related quality of life comorbidity index | | | | | | | |
